# The neural bases of the multiplication problem-size effect across countries

**DOI:** 10.3389/fnhum.2013.00189

**Published:** 2013-05-13

**Authors:** Jérôme Prado, Jiayan Lu, Li Liu, Qi Dong, Xinlin Zhou, James R. Booth

**Affiliations:** ^1^Department of Communication Sciences and Disorders, Northwestern UniversityEvanston, IL, USA; ^2^Laboratoire Langage, Cerveau et Cognition (L2C2), Centre National de la Recherche Scientifique (CNRS)Bron, France; ^3^National Key Lab of Cognitive Neuroscience and Learning, Beijing Normal UniversityBeijing, China

**Keywords:** education, arithmetic, problem-size, fMRI, multiplication

## Abstract

Multiplication problems involving large numbers (e.g., 9 × 8) are more difficult to solve than problems involving small numbers (e.g., 2 × 3). Behavioral research indicates that this problem-size effect might be due to different factors across countries and educational systems. However, there is no neuroimaging evidence supporting this hypothesis. Here, we compared the neural correlates of the multiplication problem-size effect in adults educated in China and the United States. We found a greater neural problem-size effect in Chinese than American participants in bilateral superior temporal regions associated with phonological processing. However, we found a greater neural problem-size effect in American than Chinese participants in right intra-parietal sulcus (IPS) associated with calculation procedures. Therefore, while the multiplication problem-size effect might be a verbal retrieval effect in Chinese as compared to American participants, it may instead stem from the use of calculation procedures in American as compared to Chinese participants. Our results indicate that differences in educational practices might affect the neural bases of symbolic arithmetic.

## Introduction

In mental arithmetic, the problem-size effect describes a well-known phenomenon whereby problem difficulty increases with the numerical size of the operands (Ashcraft and Guillaume, 2009). For example, single-digit multiplication problems involving relatively large numbers (e.g., 9 × 8) take longer to solve (and are more error prone) than problems involving smaller numbers (e.g., 2 × 3). Although the problem-size effect is one of the most widely observed phenomena in the cognitive arithmetic literature, its sources remain debated (Ashcraft and Guillaume, 2009).

On the one hand, the multiplication problem-size effect might occur because answers of large problems are more difficult to retrieve from long-term memory than answers of small problems (Campbell and Graham, 1985; Siegler, 1988; Ashcraft, 1992). This may be because large problems are not frequently encountered and practiced during arithmetic learning in school (Hamann and Ashcraft, 1986). Such problems tend to be associated with several candidate answers (Campbell and Graham, 1985). For example, 8 × 6 might be associated with the correct answer (i.e., 48) but also with incorrect neighboring answers from the multiplication table (e.g., 56). Because small problems are more practiced and less likely to be associated with interfering answers, their representations may be more differentiated in memory and answers of small problems should be more easily retrieved from long-term memory than large problems.

On the other hand, the multiplication problem-size effect might result from differences in strategy choices for solving small vs. large problems (Lefevre et al., 1996; Penner-Wilger et al., 2002). Specifically, LeFevre and colleagues have argued that large problems are less frequently solved by retrieval than small problems. Rather, answers of large problems may be derived from procedural calculation algorithms, such as decomposition (e.g., 8 × 9 = 8 × 10 − 8; 6 × 4 = 5 × 4 + 4) and transformation (e.g., 3 × 8 = 8 + 8 + 8) (Dowker, 2005). Procedural strategies are typically thought to be slower and more error prone than direct retrieval. Thus, the greater use of such strategies in large vs. small problems would explain the problem-size effect.

It has been proposed that the sources of the problem-size effect might depend upon differing educational backgrounds across countries (Lefevre and Liu, 1997; Campbell and Xue, 2001; Penner-Wilger et al., 2002). This hypothesis is supported by behavioral studies showing that the effect indeed differs across countries. First, although the multiplication problem-size effect can be observed on response times (RTs) and error rates in individuals educated in China and in North America, it is significantly smaller in Chinese than North American participants (Campbell and Xue, 2001). Second, whereas adults educated in North America report using both retrieval and calculation strategies when solving single-digit multiplication problems (Lefevre et al., 1996), adults educated in China report relying almost exclusively on retrieval when solving single-digit multiplication (Campbell and Xue, 2001). Third, the distributions of response times associated with small and large multiplication problems significantly differ between North American and Chinese adults, suggesting differences in strategy choices between groups (i.e., mixture of retrieval and calculation for North Americans and exclusive retrieval for Chinese) (Penner-Wilger et al., 2002). Overall, these behavioral results suggest that the source of the problem-size effect might depend upon factors associated with educational background across countries. Specifically, while the effect might be explained by differences in retrieval effort in Chinese adults, it might arise from differences in the use of calculation strategies in North American participants (Penner-Wilger et al., 2002).

Such behavioral results based on self-report and analyses of response times have been challenged, however. Specifically, it has been argued that verbal reports might be misleading because they are likely to be influenced by instructions (Kirk and Ashcraft, 2001) and might not accurately distinguish between calculation and retrieval (Fayol and Thevenot, 2012). Furthermore, because calculation procedures can be highly practiced and automatized, these might be implemented as fast as retrieval (Fayol and Thevenot, 2012). Therefore, analyzes of response times might not necessarily give meaningful insight into the strategies used in arithmetic problem-solving.

The goal of the present study was to provide additional evidence for the dependency of the problem-size effect on educational background by comparing its neural correlates in Chinese and American adults. Specifically, we used functional magnetic resonance imaging (fMRI) to measure the brain activity of adults educated in China and the United States while they evaluated small and large single-digit multiplication problems. Previous neuroimaging studies suggest that arithmetic processing relies on a heterogeneous brain network. On the one hand, left temporo-parietal regions are typically activated when problems are more likely to rely on fact retrieval, as is the case for single-digit multiplication (Lee, 2000; Andres et al., 2011, 2012; Prado et al., 2011), small problems (Stanescu-Cosson et al., 2000; Zhou et al., 2007; Jost et al., 2009; De Smedt et al., 2011), extensively trained problems (Zamarian et al., 2009), problems self-reported to be retrieved (Grabner et al., 2009), and exact arithmetic (Dehaene et al., 1999; Venkatraman et al., 2006). These regions are thought to support the verbal representation of math facts and include the left middle/superior temporal gyrus (Sandrini et al., 2003; Ischebeck et al., 2007; Zhou et al., 2007; Andres et al., 2011, 2012; Prado et al., 2011) and the left angular gyrus (Grabner et al., 2009, 2013; Zamarian et al., 2009). On the other hand, a dorsal fronto-parietal network is typically engaged when problems are more likely to involve the manipulation of numerical quantities, as is the case for single-digit subtraction (Lee, 2000; Piazza et al., 2007; Prado et al., 2011), large problems (Stanescu-Cosson et al., 2000; Zhou et al., 2007; Jost et al., 2009; De Smedt et al., 2011), untrained problems (Zamarian et al., 2009), problems self-reported to be calculated (Grabner et al., 2009), and approximate arithmetic (Dehaene et al., 1999; Venkatraman et al., 2006). This network involves the IPS, a region thought to be involved in the representation of numerical magnitudes (Nieder and Dehaene, 2009). It also involves the lateral and medial frontal cortices, which are thought to reflect the demands in working-memory and executive control associated with the manipulation of numbers (Ansari, 2008; Jost et al., 2009). Recently, these findings have been confirmed by a quantitative meta-analysis of the neuroimaging literature (Arsalidou and Taylor, 2011). This meta-analysis indicated that there are substantive overlap between the neural bases of numerical processing and arithmetic in the parietal and frontal cortices, suggesting that procedural strategies relying on numerical manipulation are likely to be used during arithmetic calculation. However, this meta-analysis also indicated that left temporal regions are specifically engaged during operations that are likely to rely on verbal fact retrieval, such as multiplication.

Overall, neuroimaging studies conducted on western adults (Stanescu-Cosson et al., 2000; Jost et al., 2009) and children (De Smedt et al., 2011) have observed a neural problem-size effect (i.e., greater activity for large than small problems) in the dorsal fronto-parietal regions typically involved in numerical calculation. While these findings suggest that the effect might stem from the greater use of calculation procedures in large than small problems, it is possible that this result might depend upon differences in cultural and educational background. To our knowledge, only two previous studies have investigated the neural correlates of arithmetic across Chinese and English languages (Tang et al., 2006; Venkatraman et al., 2006). First, by studying English–Chinese bilinguals, Venkatraman et al. (2006) found that solving arithmetic problems in a language different from the one used to learn them is associated with enhanced activity in several brain regions. These increases are observed in regions associated with verbal retrieval for exact arithmetic and regions associated with numerical manipulation for approximate arithmetic. Although this study supports the idea that regions involved in verbal retrieval and numerical processing are differentially engaged in arithmetic, it could not evaluate the effect of cultural and educational background on the neural bases of arithmetic as it focused on the same group of bilingual individuals. Second, Tang et al. (2006) recently compared the neural correlates of simple arithmetic processing in participants educated in China and Western countries. However, this study only did so with single-digit addition, and did not further dissociate between small and large problems. Because single-digit addition and multiplication diverge in terms of learning methods (Dehaene et al., 2003) and problem-solving strategies (Fayol and Thevenot, 2012), differences in the neural bases of the multiplication problem-size effect between Chinese and Western individuals remain unknown.

In the present study, we expected the neural bases of the multiplication problem-size effect to specifically differ between Chinese and Americans. Behavioral studies suggest that the problem-size effect preferentially may result from differences in retrieval effort in Chinese, whereas it preferentially may rely on differences in the use of calculation procedures in North Americans (Penner-Wilger et al., 2002). Therefore, we expected that the problem-size effect would be more strongly associated with activity in brain regions involved in the verbal representation of math facts (i.e., left mid-superior temporal gyrus and/or left angular gyrus) in Chinese as compared to American participants. Conversely, we hypothesized that the problem-size effect would be more strongly associated with activity in brain regions involved in numerical manipulation and arithmetic calculation (i.e., IPS and frontal regions) in American as compared to Chinese participants. As is common in the neuroimaging literature (Poldrack, 2006), most studies have indirectly inferred the role of temporal and parietal brain regions involved in arithmetic based on anatomical landmarks and prior research. It is increasingly believed, however, that such “reverse” inferences can be greatly strengthened by systematically localizing the cognitive processes of interest in each participant (Saxe et al., 2006). In the present study, we used independent localizer scans to identify the parietal and temporal cortices involved in verbal and numerical processing. This enabled us to improve the specificity and selectivity of our analyses.

## Materials and methods

### Participants

Thirty-two Chinese participants were recruited from the Beijing community in China, and 33 American participants were recruited from the Chicago community in the United States. Data from three Chinese and four American participants were excluded due to excessive movement in the scanner (i.e., greater than 3 mm). Two Chinese and three American participants were further excluded because their error rates were above 30%. Therefore, 27 Chinese participants [13 males; mean age = 24.2 years; standard deviation = 2.12; age range: 20–28 years] and 26 American participants [10 males; mean age = 25.2 years; standard deviation = 3.07; age range: 19–30 years] were included in the analyses. Chinese participants were native Chinese speakers, while American participants were native English speakers. All participants had a minimum of 13 years of education, which they completed in their respective countries (i.e., China or the United States). Although all participants were graduates from high-school, they varied regarding the number of years of post-secondary education they received. However, as emphasized by Campbell and Xue (2001) and Lefevre and Liu (1997), basic arithmetic skills such as single-digit multiplication are acquired and consolidated primarily during elementary education. Therefore, with respect to single-digit multiplication skill, it is unlikely that this variability in the number of years of post-secondary education might have affected our results. Nonetheless, to ensure that any fMRI differences between the Chinese and American groups were not driven by differences in math proficiency, we performed control analyses in which relevant effects were controlled for differences in multiplication skill (see below).

None of the Chinese participants were of Western descent and none of the American participants were of Asian descent. All subjects were right-handed and had no history of neurological or psychiatric disorders. Experimental protocols were approved by the local Institutional Review Boards, and informed consent was obtained from each participant. Chinese and American participants were compensated 75 RMB and 20 USD per hour for their time, respectively. Groups were comparable in terms of age (*t*_(51)_ = 1.34, *p* = 0.19) and gender (Fisher's Exact test: *p* = 0.58). The exact same individuals participated in the localizer and the arithmetic tasks.

### Task

**Figure 1:**
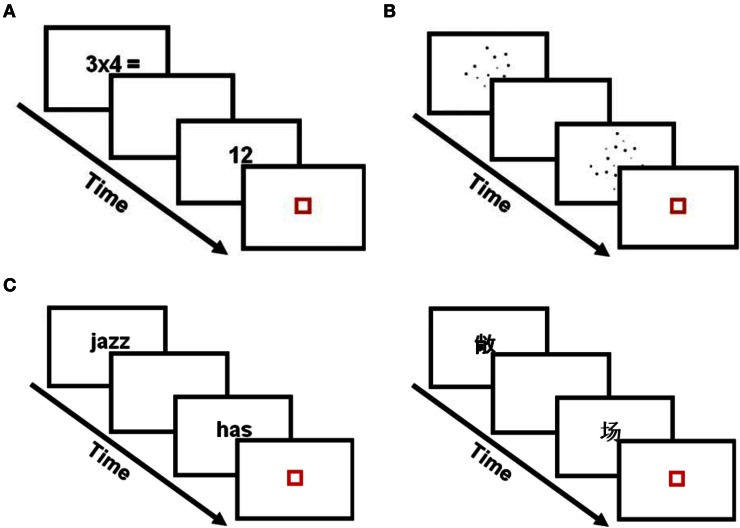
**Experimental and localizer tasks. (A)** In the multiplication task, participants were asked to evaluate the answer of single-digit multiplication problems. Problem-sizes were either small (e.g., 3 × 4) or large (e.g., 6 × 7). **(B)** In the numerical processing localizer, participants decided which of two dot arrays were composed of the larger number of dots. **(C)** In the verbal processing localizer, American participants decided whether two visually presented English words rhymed or not (left) and Chinese participants decided whether two visually presented Chinese words rhymed or not (right). In all tasks, the first stimulus was presented for 800 ms, followed by a blank screen for 200 ms. A second stimulus was then presented for 800 ms, followed by a red fixation square for 200 ms.

In each trial of the multiplication task, participants evaluated the answer of a single-digit multiplication problem involving Arabic numerals (see Figure 1A). The exact same stimuli were employed for the Chinese and American groups. Following a previous study (Prado et al., 2011), we included 12 small and 12 large multiplication problems. In small multiplication problems, the two operands were smaller than or equal to 5 (e.g., 3 × 4). In large multiplication problems, both operands were larger than 5 (e.g., 6 × 7). Each problem was repeated twice with a true answer (e.g., 3 × 4 = 12) and once with a false answer, yielding 72 trials total in each task (36 small and 36 large problems). False answers were table-related. They corresponded to the answer that would be obtained by adding or subtracting 1 to the first operand (e.g., 3 × 5 = 20 or 3 × 5 = 10). Problems involving 0 (e.g., 3 × 0), 1 as second operand (e.g., 3 × 1) and ties (e.g., 3 × 3) were not included in the main experiment but were used in the practice session. Twelve problems with a correct answer and twelve problems with a false answer were included in the practice session for each task.

### Localizer scans

Our hypotheses involved regions of the parietal and temporal cortices involved in verbal and numerical processing (see Introduction). To identify those regions and improve the sensitivity and specificity of our analyses (Saxe et al., 2006), localizer scans were included in the experiment. In the verbal processing localizer (see Figure 1C), participants decided whether two visually presented words rhymed or not. Single character (monosyllables) Chinese words were used for the Chinese group and monosyllabic English words were used for the American group. To ensure that judgments were not based solely on orthographic similarities between words, orthography and phonology were manipulated independently. That is, the two words could have similar orthography and similar phonology (e.g., dime–lime; 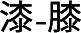; 12 trials), similar orthography but different phonology (e.g., pint–mint; 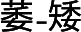; 12 trials), different orthography but similar phonology (e.g., jazz–has; 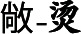; 12 trials) or different orthography and different phonology (e.g., press–list; 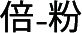; 12 trials). Similar orthography in Chinese was operationalized as sharing the same phonetic radical (right part of the character). We also included a perceptual control condition in which two symbol strings were presented on the screen instead of word pairs (12 trials). In American participants, the two symbol strings consisted of rearranged parts of lower case Courier letters. In Chinese participants, the two symbol strings were single Tibetan characters. Tibetan characters were chosen because they are similar to Chinese characters in terms of visual complexity and configuration. The perceptual condition was designed to control for visual stimulation and response selection in both groups. All participants had to determine whether the symbol strings matched (the symbols matched in half of the trials). Twelve trials of each condition were presented in the practice session. Different sets of stimuli were used in the practice and in the scanning sessions.

In the numerical processing localizer (see Figure 1B), participants decided which of two visually presented dot arrays were composed of the larger number of dots (i.e., the larger numerosity). The exact same stimuli were employed for the Chinese and American groups. The numerical comparisons were “easy” (i.e., 12 dots vs. 36 dots; 24 trials), “intermediate” (i.e., 18 dots vs. 36 dots; 24 trials), or “hard” (i.e., 24 dots vs. 36 dots; 24 trials). Six different dot sizes were used and stimuli were controlled for differences in cumulative surface areas and distribution of dot sizes (Prado et al., 2011). Twelve trials of each condition were presented in the practice session. Different stimuli were used in the practice and in the scanning sessions.

### Experimental protocol

Participants practiced the experimental and localizer tasks before entering into the scanner. In the scanner, the multiplication task and the numerosity processing localizer were decomposed in 2 functional runs of about 4 min each. The verbal processing localizer was administered in one single run lasting approximately 7 min. Participants also performed an additional subtraction evaluation task in the scanner. The data from this task will not be considered in this report. The order of the tasks was fully counterbalanced across participants. The timing and order of trial presentation within each run was optimized for estimation efficiency using optseq2 (http://surfer.nmr.mgh.harvard.edu/optseq/). Specifically, although trials appeared to be presented in a random order to participants, the timing and order of trials in each condition was calculated by optseq2 in order to remove the overlap from the estimate of the hemodynamic response (by introducing variable periods of fixation, or jitters). Behavioral responses were recorded using an MR-compatible keypad placed below the right hand. Visual stimuli were generated using E-prime software (Psychology Software Tools, Pittsburgh, PA) and projected onto a translucent screen that was viewed by the participants through a mirror attached to the head-coil.

Stimulus timing was identical in all tasks. A trial started with the presentation of a first stimulus (multiplication, dot array or word) for 800 ms, followed by a blank screen for 200 ms. A second stimulus (multiplication answer, dot array or word) was then presented for 800 ms. This second stimulus was followed by a red fixation square (duration: 200 ms) that indicated the need to make a response during an interval ranging from 2800 to 3600 ms. Twenty-four null trials were included in the multiplication task and the numerical localizer scan. Twelve null trials were included in the verbal localizer scan. In the null trials, a black square was presented for the same stimulus duration as in the experimental trials and participants were asked to press a button when the black square turned red.

### Imaging procedures

Data from the Chinese participants were collected at the State Key Lab of Cognitive Neuroscience and Learning at Beijing Normal University in China. Data from the American participants were collected at the Northwestern University's Center for Advanced MRI (CAMRI) in the United States. At both sites, the exact same scanner model (Siemens 3T TIM Trio MRI scanner; Siemens Healthcare, Erlangen, Germany) and exact same scanning parameters were used. The fMRI blood oxygenation level dependent (BOLD) signal was measured with a susceptibility weighted single-shot echo planar imaging (EPI) sequence. The following parameters were used: TE = 20 ms, flip angle = 80°, matrix size = 128 × 120, field of view = 220 × 206.25 mm, slice thickness = 3 mm (0.48 mm gap), number of slices = 32, TR = 2000 ms. Before functional image acquisition, a high resolution T1 weighted 3D structural image was acquired for each subject (TR = 1570 ms, TE = 3.36 ms, matrix size = 256 × 256, field of view = 240 mm, slice thickness = 1 mm, number of slices = 160).

### Behavioral data analysis

Behavioral studies have found that large multiplication problems were associated with both longer RT and higher error rates than small problems (Ashcraft and Guillaume, 2009). Errors, however, are known to elicit specific activity in brain regions and this may bias fMRI analyses (Holroyd and Coles, 2002). Therefore, only correct trials are analyzed in the present study and the behavioral multiplication problem-size effect is measured in terms of a difference in RT rather than error rate. Specifically, the behavioral multiplication problem-size effect was investigated by analyzing RT data on correct trials as a function of problem-size and group. This was done using a 2 × 2 ANOVA with the within-subject factor Problem-size (small, large) and the between-subject factor Group (Chinese, American).

### fMRI data analysis

Data analysis was performed using SPM5 (Statistical Parametric Mapping) (www.fil.ion.ucl.ac.uk/spm). The first six images of each run were discarded to allow for T1 equilibration effects. The remaining functional images were corrected for slice acquisition delays, spatially realigned to the first image of the first run to correct for head movements, co-registered with the segmented anatomical image, normalized to the standard T1 Montreal Neurological Institute (MNI) template volume (normalized voxel size, 2 × 2 × 4 mm^3^), and spatially smoothed with a Gaussian filter equal to twice the voxel size (4 × 4 × 8 mm^3^ full width at half maximum). The quality of the normalization was verified in each participant by visually checking the registration and ensuring an adequate correspondence between each individual's brain and the MNI template. Event-related statistical analysis was performed according to the general linear model. Trials in which an incorrect response was recorded were excluded from the analyses. Activation was modeled as epochs with onsets time-locked to the presentation of the first stimulus and with a duration of 2 s. Only hits (i.e., correct responses in problems with a true answer) were considered of interest in the behavioral and fMRI analyses of the multiplication task. All epochs were convolved with a canonical hemodynamic response function. The time series data were high-pass filtered (1/128 Hz), and serial correlations were corrected using an autoregressive AR (1) model.

Previous behavioral studies have found that the multiplication problem-size effect is larger for American than Chinese participants (Campbell and Xue, 2001). Such a difference in task performance might introduce a potential confound in the fMRI analysis because any group differences in activity could be potentially explained by this discrepancy (Church et al., 2010). To minimize this confound, we matched the Chinese and American groups in terms of their behavioral problem-size effect. Specifically, we iteratively removed from the fMRI analyses the Chinese participants with the smallest multiplication problem-size effect and the American participants with the largest multiplication problem-size effect until no significant difference was observed between groups. This procedure yielded two groups with 22 participants in each for the fMRI analyses (i.e., 44 participants total). To determine the neural correlates of the multiplication problem-size effect in these remaining participants, we calculated for each subject the contrast of large vs. small problems (i.e., the neural problem-size effect). The resulting individual contrast images were entered into two random effect (RFX) analyses: a one-sample *t*-test across all participants (Chinese and Americans) and a two-sample *t*-test coding each group separately. In both analyses, the mean-centered individual behavioral problem-size-effects were included as covariates to control for any remaining behavioral differences between groups. These analyses allowed us to identify (1) the voxels showing a significant neural problem-size effect across groups and between groups, as well as (2) the voxels whose difference in activity between large and small problems (i.e., the neural problem-size effect) co-varied with the difference in RT between large and small problems (i.e., the behavioral problem-size effect) across groups and between groups. In the localizer scans, we calculated for each participant the contrasts of (1) word pairs vs. symbol strings in the verbal processing localizer (word pairs > strings) and (2) hard vs. easy numerical comparisons in the numerical processing localizer (hard > easy). The resulting individual contrast images were subsequently entered into RFX one-sample *t*-tests.

Unless otherwise noted, group-level statistical tests were controlled for a family-wise error (FWE) rate of *p* < 0.05 across the whole brain, via a combination of individual voxel threshold of *p* < 0.005 and cluster extent threshold of 880 mm^3^ (i.e., 55 voxels). The cluster extent threshold was determined by Monte Carlo simulations (5000 iterations) conducted using the “AlphaSim” program (http://afni.nimh.nih.gov/pub/dist/doc/manual/AlphaSim.pdf) using an estimate of the smoothness of the data provided by SPM. Additionally, when no significant effect was present at this threshold, activations were examined with a FWE rate of *p* < 0.1 (across the whole brain). This was achieved by using a more lenient individual voxel threshold of *p* < 0.01 and a cluster extent threshold of 1120 mm^3^ (i.e., 70 voxels) (estimated by AlphaSim). Such a more lenient threshold allows for an examination of more diffuse activations (Hasson et al., 2007) and indicates a statistical tendency. It is thus more informative than uncorrected thresholds because it allows for an interpretation of the results while giving a precise idea about the rate of false positive (Bennett et al., 2009).

In addition, small volume corrections were applied to *a priori* regions of interest of the parietal and temporal cortex identified in the localizer scans. These were the right IPS identified in the numerical processing localizer (*x* = 36, *y* = −48, *z* = 47) and the left Middle Temporal Gyrus (MTG) identified in the verbal processing localizer (*x* = −28, *y* = −58, *z* = 21). For these two regions, activation was controlled for a FWE rate of *p* < 0.05 within a 12-mm radius sphere around each set of coordinates, via a combination of individual voxel threshold of *p* < 0.005 and cluster extent threshold of 128 mm^3^ (i.e., 8 voxels) (using AlphaSim and the procedure detailed above). All coordinates are reported in MNI space. For anatomical localization, we performed a non-linear transformation from MNI to Talairach coordinates (Talairach and Tournoux, 1988) and identified the regions activated via the Talairach Daemon software (http://ric.uthscsa.edu/resources). Cross validations were performed by overlaying each map on anatomical reference images from the Brodmann and AAL (automatic anatomic labeling) maps included in the Mricron software (www.sph.sc.edu/comd/rorden/MRIcron/).

Brain activity in activated clusters was extracted for visualization using the SPM toolbox Marsbar (http://marsbar.sourceforge.net/). Regions of Interests (ROIs) included all voxels within the activated cluster. For each participant, we calculated the average activity for each trial type within an ROI by averaging the fMRI signal across all voxels within that ROI.

## Results

### Behavior

#### Multiplication task

A main effect of Problem-size revealed that participants were slower at evaluating large than small multiplication problems [*F*_(1, 51)_ = 29.23, MSE = 8, 554, *p* < 0.00001]. Therefore, a significant behavioral problem-size effect (i.e., difference in RT between large and small problems) was observed across all participants. However, this effect interacted with Group [*F*_(1, 51)_ = 6.67, MSE = 8, 554, *p* = 0.013], such that the problem-size effect was greater in American than Chinese participants (144 ms for American participants, 52 ms for Chinese participants). This was the case despite the fact that the problem-size effect was significant in each group separately [Chinese: *t*_(26)_ = 4.02, *p* = 0.0004; American: *t*_(25)_ = 4.23, *p* = 0.0003]. Finally, the ANOVA revealed a main effect of Group [*F*_(1, 51)_ = 51.97, MSE = 63, 026, *p* < 0.00001], indicating that Chinese participants were faster than American participants.

**Figure 2:**
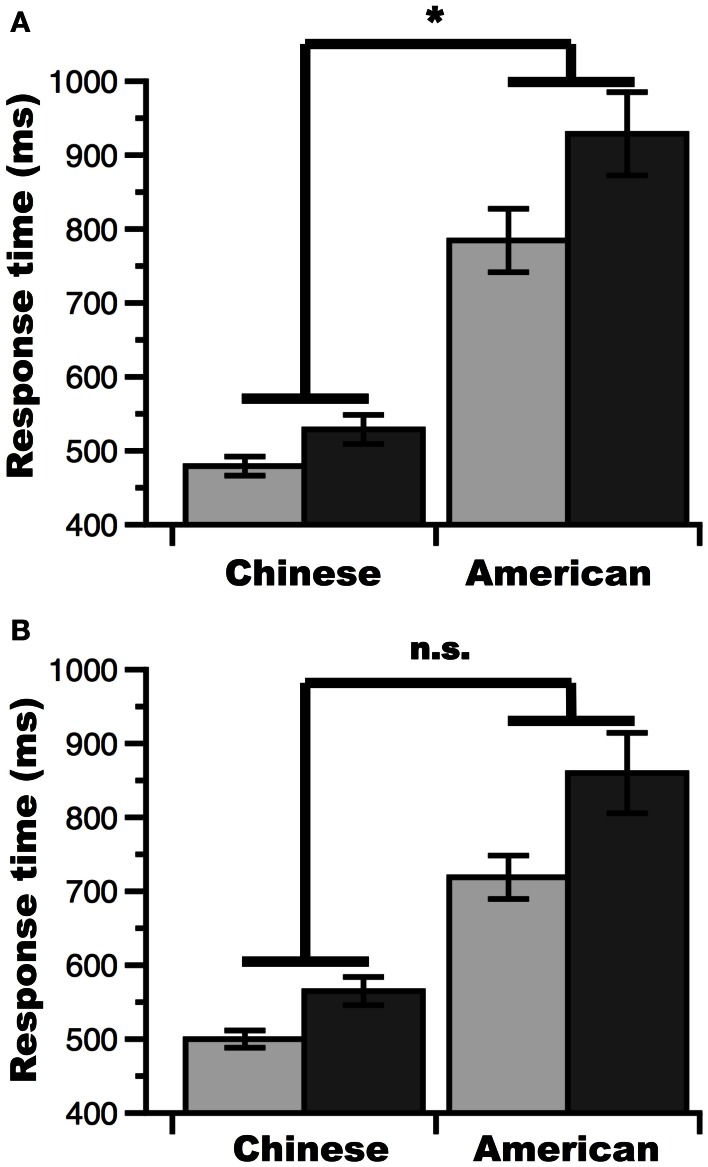
**Behavioral performance on the multiplication task. (A)** Before equating for the magnitude of the problem-size effect between groups, the multiplication problem-size effect was significantly larger for American than Chinese participants. **(B)** After equating for the magnitude of the problem-size effect between groups, the multiplication problem-size effect was no longer significantly larger for American than Chinese participants. light gray, small problems; dark gray, large problems; ^*^p < 0.05; n.s., non-significant; error bars, standard error of the mean.

In line with previous findings (Campbell and Xue, 2001), our results indicate that the behavioral multiplication problem-size effect was larger in American than Chinese participants. To minimize this behavioral confound in fMRI analyses, we attempted to match the Chinese and American groups in terms of their behavioral problem-size effect (see Materials and Methods). After the matching procedure, the multiplication problem-size effect was still significant across participants [Chinese: *F*_(1, 21)_ = 16.24, MSE = 2, 482, *p* = 0.0006; American: *F*_(1, 21)_ = 14.42, MSE = 15, 109, *p* = 0.002] (see Figure 2). However, the interaction between Group and multiplication Problem-size effect was no longer significant [*F*_(1, 42)_ = 4.05, MSE = 8796, *p* = 0.051], indicating that the problem-size effect was more comparable across groups (although there remained a numerical difference between the problem-size effect between the groups).

#### Localizer scans

In the verbal processing localizer, mean RT for correct responses was submitted to a 2 × 2 ANOVA with the within-subject factor Stimulus type (word pairs, symbol strings) and the between-subject factor Group (Chinese, American). The ANOVA revealed a main effect of Stimulus type [*F*_(1, 51)_ = 47.87, MSE = 8142, *p* < 0.00001], a main effect of Group [*F*_(1, 51)_ = 24.03, MSE = 114, 762, *p* = 0.00001] and an interaction of Stimulus type and Group [*F*_(1, 51)_ = 44.81, MSE = 8142, *p* < 0.00001]. Therefore, although participants took overall longer to evaluate word pairs than symbol strings, the effect was greater in Chinese (926 ms vs. 689 ms) than in American adults (1143 ms vs. 1137 ms).

In the numerical processing localizer, mean RT for correct responses was submitted to a 2 × 2 ANOVA with the within-subject factor Comparison difficulty (easy, intermediate, hard) and the between-subject factor Group (Chinese, American). We found a main effect of Comparison difficulty [*F*_(2, 102)_ = 42.82, MSE = 2410, *p* < 0.00001], indicating that RT increased as comparison difficulty increased (easy: 734 ms, intermediate: 760 ms, hard: 820 ms). Although the ANOVA revealed faster RT for Chinese than American participants [*F*_(1, 51)_ = 28.13, MSE = 246, 022, *p* < 0.00001], there was no interaction between Group and Comparison difficulty [*F*_(2, 102)_ = 0.79, MSE = 2410, *p* = 0.46]. Thus, consistent with previous research (Pinel et al., 2001; Prado et al., 2011), there was an inverse relationship between RT and the numerical distance between numerosities of the dot patterns (i.e., a distance effect). This effect, however, was not modulated by group.

Finally, the size of the main effect of Comparison difficulty in the numerical processing task was comparable to the size of the main effect of Stimulus type in the verbal processing localizer [86 ms vs. 121 ms, *t*_(52)_ = 1.41, *p* = 0.16]. Therefore, both localizer contrasts were comparable in terms of difficulty.

### fMRI results

#### Localizer scans

**Figure 3:**
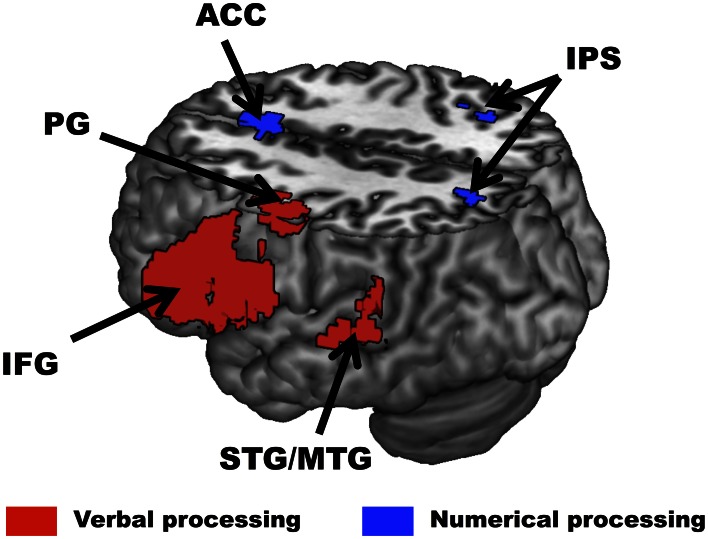
**Brain regions identified by the localizer scans across all participants.** In the verbal processing localizer (red), greater activity for words than symbols was observed in the left Superior and Middle Temporal Gyri (STG/MTG), left Inferior Frontal Gyrus (IFG), and left Precentral Gyrus (PG). In the numerical processing localizer (blue), greater activity for difficult than easy comparisons was observed in the right Intraparietal Sulcus (IPS) and Anterior Cingulate Cortex (ACC). All activations are overlaid on a 3D rendering of the MNI-normalized anatomical brain. The upper part of the brain is cut out (*Z* = 46) to show activations in deeper sulci and along the medial wall of the cortex.

As described in the Materials and Methods, localizer scans served to identify *a priori* regions of interest of the parietal and temporal cortices (i.e., IPS and MTG) involved in verbal and numerical processing for small volume correction of the main analyses. Across all participants, the verbal processing localizer identified a region of the left mid-superior temporal cortex more active for words than symbol strings (*x* = −28, *y* = −53, *z* = 21). Additional activation was observed in dorsal and ventral parts of the left Inferior Frontal Gyrus (IFG), left Middle Frontal Gyrus (MFG) and left Precentral Gyrus (PG) (see Figure 3 and Table 1 for a full list of activated regions). In the numerical processing localizer, enhanced activity was observed for hard than easy comparisons in the right IPS (*x* = 36, *y* = −48, *z* = 47). Additional activation was observed in a fronto-parietal network encompassing the left Precuneus, left ventral IFG, and Anterior Cingulate Cortex (ACC) (see Figure 3 and Table 1 for a full list of activated regions).

**Table 1:** **Clusters activated in the localizer scans across all participants**.

**Anatomical regions**	**~BA**	**Cluster size (mm**^**3**^**)**	**MNI coordinates**			***Z* scores**
			***X***	***Y***	***Z***	
**VERBAL PROCESSING LOCALIZER (WORDS > SYMBOLS)**						
L. Inferior/Middle frontal gyrus	46/47	21200	−44	20	21	5.44
L. Middle temporal gyrus	21	1648	−28	−53	21	3.46
R. Caudate	–	7696	36	−43	2	4.86
L. Precentral gyrus	6	2336	−48	−3	48	4.62
L. Caudate	–	1600	−4	1	18	4.09
L. Middle/superior temporal gyrus	21/22	1952	−55	−37	6	3.91
L. Parahippocampal gyrus	28	976	−24	−18	−13	3.88
L. Anterior cingulate gyrus	25	880	−4	19	−4	3.85
L. Cuneus	18	2528	0	−73	15	3.24
R. Middle frontal gyrus	9	21200	4	50	23	3.11
**NUMERICAL PROCESSING LOCALIZER (HARD > EASY COMPARISONS)**						
R. Inferior frontal gyrus	9	4704	50	9	25	5.18
R. Anterior cingulate gyrus	32	896	10	21	39	3.83
L. Precuneus/superior parietal lobule	7	920	−18	−56	51	3.9
R. Intra-parietal sulcus	40	904	36	−48	47	3.78

Notes. All clusters survive a threshold of p < 0.05 FWE corrected for multiple comparisons.

L, left; R, right; ~BA, approximate Brodmann Area; MNI, Montreal Neurological Institute.

#### Multiplication problem-size effect

Across Chinese and American participants, a significant neural problem-size effect (i.e., greater activity for large than small multiplication problems) was observed in several fronto-parietal regions, including the left IPS, bilateral IFG, left MFG, and ACC (see Table 2). Critically, however, the effect differed between groups.

**Table 2:** **Clusters showing a multiplication neural problem-size effect**.

**Anatomical regions**	**~BA**	**Cluster size (mm**^**3**^**)**	**MNI coordinates**			***Z* scores**
			***X***	***Y***	***Z***	
**ACROSS ALL PARTICIPANTS (LARGE > SMALL)**						
R. Insula	13	4592	34	20	4	5.62
L. Inferior frontal gyrus	47	13504	−40	18	−12	5.56
L. Precuneus/intraparietal sulcus	7/40	7328	−30	−46	44	4.75
R. Medial frontal gyrus	6	1936	0	14	52	4.42
L. Cuneus	18	2880	0	−80	24	4.22
**CHINESE (LARGE > SMALL) > AMERICAN (LARGE > SMALL)**						
L. Paracentral lobule/precuneus	5/7	14112	−10	−44	60	5.14
R. Superior temporal gyrus	42	1616	58	−24	4	4.68
R. Medial frontal gyrus	11	3360	6	48	−12	4.31
L. Superior temporal gyrus	22	1488	−64	−16	4	3.96
L. Precentral/postcentral gyrus	4/3	944	−54	−16	44	3.72
L. Insula	13	896	−38	−24	20	3.6
R. Fusiform gyrus	19	896	24	−60	−8	3.06
**AMERICAN (LARGE > SMALL) > CHINESE (LARGE > SMALL)**						
L. Medial frontal gyrus	8	1968	−4	20	52	3.59
R. Intraparietal sulcus^*^	40	128	32	−60	44	2.98

Notes. All clusters survive a threshold of p < 0.05 FWE corrected for multiple comparisons.

L, left; R, right; ~BA, approximate Brodmann Area; MNI, Montreal Neurological Institute.

* Region significantly activated after small volume correction.

**Figure 4:**
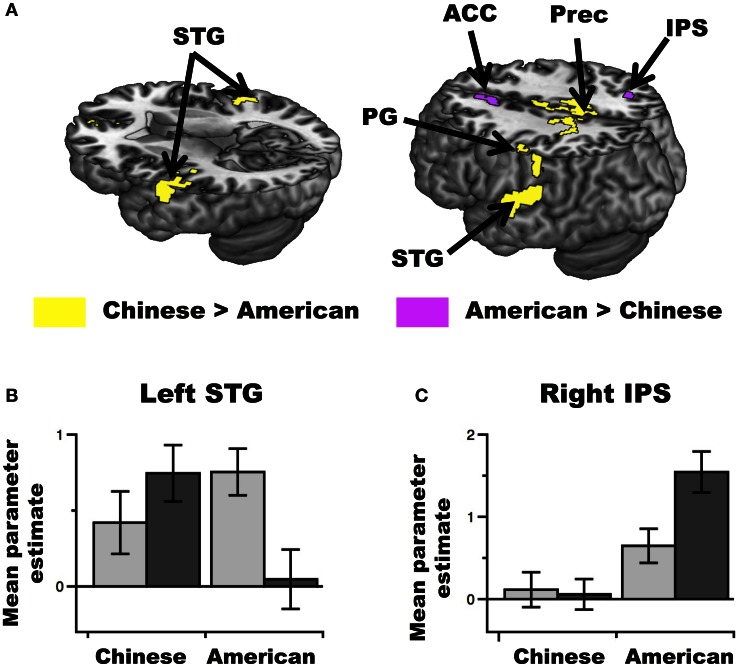
**Group differences in the neural problem-size effect (i.e., difference in activity between large and small multiplication problems). (A)** A greater neural problem-size effect was observed in Chinese than American participants in the bilateral Superior Temporal Gyrus (STG), left Postcentral Gysrus (PG), and Precuneus (Prec) (yellow). A greater neural problem-size effect was observed in American than Chinese participants in the right Intraparietal Sulcus (IPS) and Anterior Cingulate Cortex (ACC) (purple). All activations are overlaid on 3D renderings of the MNI-normalized anatomical brain. The upper part of the brain is cut out at two different heights (left panel: *Z* = 8; right panel: *Z* = 46) to show activations in deeper sulci and along the medial wall of the cortex. **(B)** Plot of the brain activity observed for large (dark gray) and small (light gray) problems as a function of group in the left STG cluster (for visualization only). **(C)** Right: Plot of the brain activity observed for large (dark gray) and small (light gray) problems as a function of group in the right IPS cluster (for visualization only). Note that the scale is different than that in **(B)**.

First, we found a greater neural problem-size effect for Chinese than American participants in the bilateral Superior Temporal Gyrus (STG), as well as in the left precentral/postcentral gyri and precuneus (see Figure 4A and Table 2). A visualization of the pattern of brain activity in the left and right STG revealed that the group difference was driven by a positive neural problem-size effect in Chinese participants, and a negative effect for American participants (see Figure 4B for a plot in the left STG). The left MTG/STG cluster identified in the verbal processing localizer did not overlap with the left STG cluster exhibiting the group difference in neural problem-size effect. However, overlap was observed in the left STG at a FWE corrected threshold of *p* < 0.1, indicating a statistical tendency.

Second, we found a greater neural problem-size effect for American than Chinese participants in the right IPS and ACC (activation in the right IPS was found after small volume correction based on the peak activity obtained in the numerical localizer task, see Materials and Methods) (see Figure 4A and Table 2). The peak activity of this right IPS cluster was less than 12 mm away from the peak coordinates of the right IPS region identified in the numerical processing localizer. In the IPS, an examination of the pattern of brain activity revealed that the group difference was driven by a larger positive neural problem-size effect for American than Chinese participants (see Figure 4C).

Simple effect analyses were then conducted to assess the significance of the neural problem-size effect in each group separately. First, in both the right IPS (*x* = 30, *y* = −60, *z* = 36; *Z* = 3.77) and the ACC (*x* = −2, *y* = 24, *z* = 36; *Z* = 4.09), we found a significant neural problem-size effect across American participants. Importantly, the effect in the IPS was absent in Chinese participants. Second, we did not find a significant neural problem-size effect across Chinese participants in either the left or right STG at our stringent FWE corrected threshold of *p* < 0.05. However, this effect tended to be significant in both of these regions, as revealed by further analyses conducted at a threshold of *p* < 0.1 (FWE corrected across the whole-brain). Furthermore, small multiplication problems tended to be associated with more activity than large multiplication problems (i.e., a reverse neural problem-size effect) in American participants (*p* < 0.1 FWE corrected) in both of these regions.

#### Individual differences in the multiplication problem-size effect

**Figure 5:**
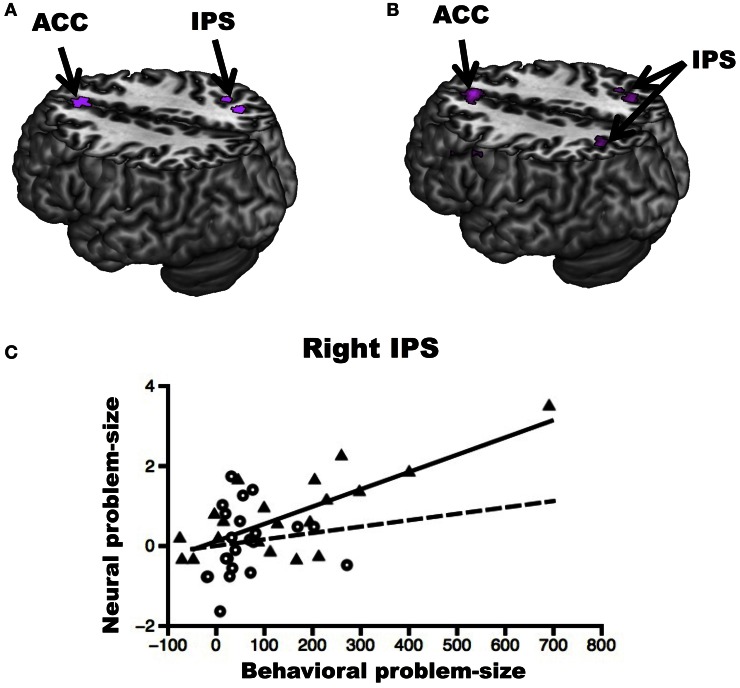
**Inter-individual variability in the multiplication problem-size effect. (A)** Across American participants, a larger behavioral problem-size effect (difference in response time between large and small problems) was associated with a larger neural problem-size effect (difference in brain activity between large and small problems) in the right Intraparietal Sulcus (IPS) and the Anterior Cingulate Cortex (ACC). **(B)** A stronger positive relationship between behavioral and neural problem-size effects was observed in American than Chinese participants in the bilateral IPS and ACC. **(C)** Plot of the relationship between the behavioral and neural problem-size effect in the right IPS for American (triangles, solid line) and Chinese (circles, dotted line) participants (for visualization only). Brain activity was extracted in the cluster showing a stronger positive relationship between behavioral and neural problem-size effects in American than Chinese participants for multiplication. The relationships between behavioral and neural problem-size effects remained significant in the right IPS and ACC when the two subjects with the largest behavioral effects were removed from the analysis. All activations are overlaid on a 3D rendering of the MNI-normalized anatomical brain. The upper part of the brain is cut out (*Z* = 46) to show activations in deeper sulci and along the medial wall of the cortex.

Overall, the results above suggest that the neural sources of the multiplication problem-size effect differ in Chinese and American participants. To test whether activity in the brain regions found above was related to behavioral performance, we then identified the voxels in which there was a reliable between-subject relationship between the behavioral and neural problem-size effects in Chinese and American participants across the whole-brain. Although we did not find any regions showing such a relationship across Chinese participants, we found that a larger neural problem-size effect was associated with a larger behavioral problem-size effect across American participants in the right IPS, ACC and right IFG (see Figure 5A). Critically, this relationship was more positive across American than Chinese participants in all of these regions (see Figure 5B and Figure 5C for a plot in the right IPS). Therefore, the neural bases of inter-individual variations in the problem-size effect also differed between the Chinese and American group.

#### Control analyses

The group differences in the neural problem-size reported above are consistent with our hypotheses. However, it is important to ensure that such effects are not driven by other factors.

First, consistent with previous reports (Lefevre and Liu, 1997; Campbell and Xue, 2001), American participants were less proficient in single-digit multiplication than Chinese participants: they displayed poorer overall performance and a larger problem-size effect. We controlled for group differences in behavioral problem-size effect by including this factor as covariate in our main analyses and by matching the groups in terms of this effect. To further rule out the possibility that overall group differences in multiplication performance were driving our results, we performed an additional set of analyses in which we included both problem-size effect and overall response time as nuisance covariates. The results obtained with this model were similar to the results obtained in our initial analysis. Specifically, all the clusters in which we observed differences in the neural problem-size effect in Chinese vs. Americans remained significant with these covariates. This suggests that none of our results were due to differences in overall performance between groups.

Second, as can be seen on Figure 5C, inter-individual differences in the behavioral problem-size effect were larger in the American than in the Chinese group. Although none of the American participants can be considered outliers (defined as >3 standard deviations from the mean), it remains possible that the more positive relationship between the behavioral and neural problem-size effects in the IPS for American compared to Chinese participants might be driven by a greater inter-individual variability. Therefore, we conducted another set of analyses in which we removed the two American participants with the largest behavioral problem-size effects, thereby equating inter-individual variability between groups. Again, the results obtained with these analyses were similar to the results obtained in our initial analyses. Specifically, there was still a reliable positive relationship across American participants in the right IPS and ACC (but not in the right IFG). This relationship was also greater in Americans than Chinese in both of these regions.

Third, Chinese and American participants were scanned on two different MRI scanners. To minimize this factor, the exact same experimental protocol, scanner model, and scanning protocol were used. However, it remains possible that some of the between-group differences might still be affected by scanner-related factors (e.g., in shim or magnetic susceptibility). Importantly, such scanner-related biases should affect all contrasts to the same degree. This includes low-level contrasts in which one would not expect any differences between groups. To test for this possibility, we contrasted the brain activity associated with null trials in Chinese vs. American participants. We did not find any brain regions differentially activated between groups, even at a lenient threshold of *p* < 0.01 uncorrected. Therefore, it seems unlikely that systematic differences between scanners might have biased our results.

## Discussion

The problem-size effect is one of the most robust and consistent phenomena in the cognitive arithmetic literature (Ashcraft and Guillaume, 2009). Yet, there is a debate as to whether the effect reflects differences in retrieval effort or in the use of calculation procedures between large and small problems (Ashcraft and Guillaume, 2009). Several behavioral studies suggest that the sources of the problem-size effect might in fact depend upon differing educational backgrounds across countries (Lefevre and Liu, 1997; Campbell and Xue, 2001; Penner-Wilger et al., 2002). Specifically, while the effect might result from differences in retrieval effort in Chinese participants, it might stem from differences in the use of calculation strategies in North American participants (Penner-Wilger et al., 2002). The present fMRI study sought to test this hypothesis by investigating the neural bases of the multiplication problem-size effect in Chinese and American adults.

### Neural multiplication problem-size effect across all participants

Across all Chinese and American participants, we found greater overall activity for large than small multiplication problems in a network of dorso-parietal brain regions encompassing the IPS as well as the lateral and medial prefrontal cortex. This finding is consistent with several previous neuroimaging studies that have shown that these regions are more active for large than small problems (Stanescu-Cosson et al., 2000; Zhou et al., 2007; Jost et al., 2009; De Smedt et al., 2011). Although this finding might be interpreted as reflecting greater use of calculation procedures in large than small problems (De Smedt et al., 2011), follow-up analyses revealed that it was mostly driven by the American group. As discussed below, our study suggests that the neural bases of the multiplication problem-size effect are affected by country and educational background.

### Neural multiplication problem-size effect in Chinese participants

Our results revealed a larger neural problem-size effect in Chinese than American participants in the bilateral STG. The left STG cluster tended to overlap with the mid-superior temporal region identified in the rhyming task that was used as verbal processing localizer. Several neuropsychological (Sandrini et al., 2003; Van Harskamp et al., 2005) and neuroimaging (Zhou et al., 2007; Andres et al., 2011, 2012; Prado et al., 2011) studies have suggested that regions of the mid-superior temporal cortex (especially in the left hemisphere) are involved in the representation of math facts in verbal memory. For example, studies have found that lesions of the left mid-superior temporal regions are associated with impaired retrieval of multiplication facts (Lampl et al., 1994; Sandrini et al., 2003; Van Harskamp et al., 2005; Delazer et al., 2006). In a previous study, we have suggested that the left MTG might be involved in the storage of the semantic association between a multiplication problem and its answer (Prado et al., 2011). In the present study, the greater involvement of the left STG in Chinese than American participants is broadly consistent with a general role of the temporal cortex in lexical-semantic processing (Vandenberghe et al., 1996; Price et al., 1997; Rissman et al., 2003). However, it is interesting to note that the left STG is typically associated with phonological (rather than semantic) processing in the literature (Friederici, 2012; Wu et al., 2012) and that both the left and right STG are believed to play an important role in letter to speech sound mapping (Suzuki and Sakai, 2003; Hickok and Poeppel, 2007; Blau et al., 2009). Activation of the bilateral STG might thus also reflect the greater involvement of phonological representations during the processing of large vs. small multiplication problems in Chinese as compared to American participants. This might be due to the fact that, unlike Americans, Chinese memorize multiplication facts as rhyming formulas in school, thanks to the single-syllable structure of Chinese number words (this strategy is reflected in the name of the Chinese multiplication table, i.e., Nine Nine song).

It is interesting to note that a larger neural problem-size effect in Chinese than American participants was also observed in other brain regions, such as the precentral and postcentral gyrus and the precuneus. Such activations were not *a priori* predicted and, in the absence of any relevant localizers, must be interpreted with caution. However, the involvement of these regions may indicate that factors other than verbal retrieval might differentiate arithmetic processing in Chinese and American participants. For example, although only Arabic numerals were used in this task, reading experience may affect arithmetic processing (Tang et al., 2006). Because the Chinese writing system places greater demands on visuo-spatial processing than the English writing system, these activations might thus reflect enhanced visuo-spatial processing in Chinese participants (Tang et al., 2006; Cantlon and Brannon, 2007). Such activations might also reflect the use of alternative visuo-spatial strategies in Chinese participants, such as abacus imagery (Cantlon and Brannon, 2007).

### Neural multiplication problem-size effect in American participants

We also found a larger neural problem-size effect in American than Chinese participants in the right IPS and the ACC. The IPS is believed to house neuronal populations sensitive to numerical magnitudes (Nieder and Dehaene, 2009) and to be a critical region for numerical processing in general (Ansari, 2008). This region is consistently found activated in tasks involving numerical comparison (Ansari, 2008) and arithmetic problem-solving (Dehaene and Cohen, 2007). Critically, enhanced activity in the IPS has been observed when problems are solved with calculation procedures rather than retrieved from memory (Grabner et al., 2009). Such enhanced activation of the IPS is typically accompanied with greater recruitment of frontal regions, including the ACC (Grabner et al., 2009). Recruitment of such frontal regions has been attributed to the greater demands in working-memory and executive control associated with calculation strategies (Delazer et al., 2003). Therefore, our findings suggest that the multiplication problem-size effect might result from a greater use of calculation procedures in large vs. small problems in American as compared to Chinese participants.

### Multiplication and verbal retrieval in China

Why would the problem-size effect be more associated with differences in verbal representations in Chinese than American participants? One possibility is that the Chinese education system places greater emphasis on verbal memorization methods than American education (Zhang and Zhou, 2003). To some extent, rote verbal teaching methods are employed in both China and the United States. Multiplication tables are used to teach multiplication in Chinese and American elementary schools, but those methods tend to be used earlier and more extensively in China than in the United States (Zhang and Zhou, 2003). The result is that Chinese children spend more time practicing multiplication facts than American children, both in school and at home. Rote verbal memorization of multiplication facts in Chinese children might also be facilitated by cultural specificities. For example, the Chinese multiplication table is shorter and easier to memorize than the tables typically used in American schools (Zhou et al., 2007). Rote verbal learning is further made easier by the relative transparency and conciseness of Chinese words for numbers, as compared to English words (Miller et al., 1995). Overall, these educational divergences might explain why a greater proportion of Chinese than North American adults rely on direct retrieval strategies to solve both small and large multiplication problems (Campbell and Xue, 2001), and why the multiplication problem-size effect might be more strongly related to differences in verbal representations in Chinese as compared to American participants.

### Multiplication and calculation procedures in Americans

The greater reliance on calculation procedures in American than Chinese participants might be explained by the greater emphasis that American education tends to place on the comprehension of mathematical concepts during childhood (such as numerical magnitude or numerical order) than on verbal memorization methods *per se* (Graham and Fennell, 2001). Overall, less extensive reliance on rote verbal learning in the United States than in China is likely to lead to weaker associations between multiplication problems and their solutions in American as compared to Chinese adults, which might lead to a greater use of indirect calculation procedures. This may be especially true for problems involving large problem-sizes, which are typically less drilled in school than problems involving smaller operands (Hamann and Ashcraft, 1986). Indirect strategies used by American participants could involve decomposing a relatively large problem-size item (e.g., 9 × 8) into a multiplication problem that is easier to retrieve from memory (e.g., 10 × 8 = 80) and using a different operation to calculate the results (e.g., 80 − 8 = 72). It might also involve transforming a multiplication problem (e.g., 3 × 8) into easier addition problems (e.g., 8 + 8 + 8). These indirect strategies involve a manipulation of numerical magnitudes through addition or subtraction and are more likely to engage numerical processing mechanisms in the IPS (as well as control processes in the ACC) than verbal retrieval mechanisms in the mid-superior temporal gyrus. In keeping with these observations, we found more activity for large than small multiplication problems in American participants in the IPS and ACC, but not in any regions of the temporal cortex. Instead, we found that large problems tended to be associated with less activity than small problems in the left STG (see Figure 4B). Therefore, large problems might be more likely to be solved by backup strategies and calculation procedures than verbal retrieval in American participants. Overall, our results are consistent with the view that a failure to retrieve the answer of large problems and a more extensive use of calculation procedures in large vs. small problems might give rise to the problem-size effect in American adults (Lefevre and Liu, 1997; Penner-Wilger et al., 2002).

Interestingly, a previous study found greater activity in the left STG (as well as in the left IFG) for single-digit addition in English-speakers as compared to Chinese-speakers (Tang et al., 2006). Because this study did not categorize problems as a function of their sizes, it is impossible to know whether the effect was driven by small or large problems (or both). Nonetheless, an examination of the pattern of activity in the left STG in the present study (see Figure 4B) indicates that small multiplication problems also elicited numerically higher activity in American than Chinese participants. Therefore, the higher left STG activity for English than Chinese speakers observed by Tang et al. (2006) might have been primarily driven by small addition problems and may reflect greater retrieval effort for these problems in English speakers. Critically, although Tang et al. (2006) did not find any reliable group differences in regions associated with numerical calculation, English-speakers tended to engage more extensive activity in the right parietal cortex than Chinese-speakers (see Figure 1 in Tang et al., 2006). Our findings suggest that this effect might have been driven by large problems and might reflect a greater use of calculation procedures in English than Chinese-speakers.

### Individual differences in the multiplication problem-size effect

Further support for the greater use of calculation procedures in American than Chinese participants is given by an analysis of the inter-individual variability in the multiplication problem-size effect. We found that a larger behavioral problem-size effect was associated with a larger neural problem-size effect in both the IPS and ACC across American participants, but not across Chinese participants. Furthermore, this relationship was significantly stronger for American than Chinese participants. Therefore, even if the problem-size effect is likely to result from the use of calculation procedures in most American participants, this is especially true for participants exhibiting the largest problem-size effects. This was, however, not the case for Chinese participants. Surprisingly, we did not find any relationship between behavioral and neural multiplication problem-size effects in the left mid-superior temporal gyrus across Chinese individuals. Although it is always difficult to interpret a null effect, it is possible that this lack of relationship might be due to the smaller inter-individual variability observed in the Chinese sample than in the American sample. Future studies with a larger number of subjects and greater inter-individual variability might examine the relationship between behavioral and neural problem-size effects in Chinese participants.

### Other factors that may have influenced the between-group comparison

Although we argue that the between-group differences observed here result from divergences in educational backgrounds across countries, other potential factors should be considered. For example, between-group differences might result from differences in MRI scanners (Costafreda et al., 2007; Gountouna et al., 2010; Yendiki et al., 2010), language processing (Bolger et al., 2005) and/or performance levels (Church et al., 2010). However, none of these factors appear to provide a better explanation of our findings than differences in educational methods across countries. First, although the present data were acquired at two different sites, the exact same experimental protocol, scanner type and scanning protocol were used in both sites. Several studies have shown that, when these precautions are taken, activation variability due to scanner site is small compared to inter-individual variability in the cognitive task (Costafreda et al., 2007; Gountouna et al., 2010; Yendiki et al., 2010). Those studies all conclude that multi-site studies are reliable. Second, arithmetic problems were presented in the same Arabic numeral form to both Chinese and American participants, thus controlling for linguistic differences between groups. Third, a limitation of our study is that we did not acquire measures of intellectual and arithmetic abilities for each participant. Therefore, the differences between the Chinese and American groups might be attributable to overall differences in arithmetic skill, rather than differences in educational background. This may be problematic because differences in proficiency have been found to affect the neural bases of arithmetic processing (Grabner et al., 2007; Matejko et al., 2012; Price et al., 2013). However, this possibility is unlikely for two reasons. Firstly, behavioral research has shown that equating groups of Chinese and North-American adults for overall (multi-digit) arithmetic performance does not remove differences in single-digit multiplication performance: Chinese are still faster overall and exhibit a smaller problem-size effect than North-Americans (Lefevre and Liu, 1997). Therefore, the smaller multiplication problem-size effect observed in Chinese than American participants is more likely to be due to cultural and/or educational factors than proficiency *per se* (Lefevre and Liu, 1997). Secondly, we controlled for overall group differences in skill by (1) matching groups in terms of size of the problem-size effect and (2) including in our fMRI analyses the behavioral problem-size effect and the overall response time as nuisance covariates. Therefore, although we cannot definitely rule out the hypothesis that some of our results might be attributable to differences in proficiency, we think that the differences observed in the present study are more likely to stem from differences in educational backgrounds.

## Conclusion

In sum, our findings support the idea that the source of the multiplication problem-size effect may vary across countries (Penner-Wilger et al., 2002). Specifically, the neural dissociation observed between STG and IPS for large and small problems indicates that the effect is more likely to be due to reliance on verbal representations in Chinese than American individuals, while it might more likely result from the use of calculation procedures in American than Chinese individuals. Our direct demonstration of differences in the reliance on these underlying mechanisms in Chinese and American adults is in keeping with prior behavioral research based on self-report and analyses of reaction time (Lefevre and Liu, 1997; Campbell and Xue, 2001; Penner-Wilger et al., 2002). Together with Tang et al. (2006), our study indicates that the neural bases of elementary arithmetic are modulated by educational differences across countries. Such findings might be important for understanding the effects of different teaching methods on the neural representations of arithmetic (Dowker, 2005). They might also improve our knowledge of the neural bases of math learning disabilities across countries, as those are likely to stem from different sources (Geary, 2010) depending on education and cultural background.

### Conflict of interest statement

The authors declare that the research was conducted in the absence of any commercial or financial relationships that could be construed as a potential conflict of interest.
